# Effectiveness of theory-based educational interventions of promoting oral health among elementary school students

**DOI:** 10.1186/s12889-023-17528-0

**Published:** 2024-01-09

**Authors:** Samane Shirahmadi, Saeed Bashirian, Ali Reza Soltanian, Akram Karimi-shahanjarini, Farshid Vahdatinia

**Affiliations:** 1 Department of community oral health, School of dentistry, Dental research center, Hamadan University of Medical Sciences, Hamadan, Iran; 2 Department of Public Health, School of Public Health, Hamadan University of Medical Sciences, Hamadan, Iran; 3 Department of Biostatistics, School of Health, Hamadan University of Medical Sciences, Hamadan, Iran; 4 Dental implants research center, Hamadan University of Medical Sciences, Hamadan, Iran

**Keywords:** Oral health promotion, Social cognitive theory, Oral hygiene index, Telegram group, Gamification

## Abstract

**Background:**

The aim of the present study was to determine the effect of oral health education programs on the oral health of primary school students.

**Methods:**

In this randomized controlled trial study, 190 elementary fifth-grade female students were chosen using the multistage cluster sampling method. In this study, the Plaque Index (PI), Simplified Oral Hygiene Index (OHI-S), Community Periodontal Index (CPI), tooth brushing using fluoride toothpaste, dental flossing frequency and factors affecting them were determined according to social cognitive theory (SCT). Interventions were implemented using the play method and with the help of three pamphlets, five posters, a celebration of oral health, and the creation of a Telegram group. Data were analyzed using descriptive statistics indexes, t tests, paired sample t tests, chi-square tests, and Pearson correlation tests.

**Results:**

The results showed that 3 months after the intervention, compared to before the intervention, the percentage of participants in the intervention group who brushed their teeth twice or more per day increased by 48.5%, and the percentage of participants who used dental floss at least once per day increased by 64.2%. The rate of gum bleeding decreased by 6.3%. The good OHI-S rate increased by 44.4%. Dental plaque decreased by 38.1%.

**Conclusion:**

The results demonstrated that a gamification design can be effective and useful in promoting the oral health of students.

**Trial registration:**

registration timing: retrospective, registration date: 18/10/2022, registration number: IRCT20141128020129N2.

## Background

Tooth decay is the most common chronic infectious disease among elementary school students [1]. In addition to tooth decay, most children have signs of gingival and periodontal diseases globally [2].

Children with tooth decay and periodontal disease experience pain, discomfort, acute and chronic infections, sleep and eating disorders, missed school days and reduced learning ability. These diseases affect the nutrition, growth process, and child development by exerting a deep impact on the quality of life of children and their families [3].

Over the past years, there has been an ascending increase in the number of tooth decays [4, 5] and the prevalence of periodontal diseases [6, 7] in Iranian children. On average, Iranian school-aged children of 6–7 years of age and 12-year-olds experienced 4.3 and 1.6 decayed teeth in 2004, respectively. However, this index reached 4.5 and 1.71 in children aged 5-6 and 12 years in 2012, respectively [8, 9].

The results of the national survey of oral health in Iran showed that 6.8 and 2.7% of children aged 5-6 years had non-plaque and plaque gingivitis, respectively. On the other hand, 13.2 and 6.1% of children aged 12 years had non-plaque and plaque gingivitis, respectively [8].

Compared to the 2012 survey 9.7% of 5–6-year-olds and 26.9% of 12-year-olds had non-plaque-induced gingivitis, respectively [9], it is evident that the oral health status of some Iranian children is declining.

In Iran, dental care services are very expensive and account for the second highest healthcare expenditures following hospitalization charges [10, 11].

The lack of dental insurance coverage, governmental policy and commitment to advance oral health as public health services, and access to dental facilities in less developed parts of the country are some of the reasons that the underprivileged strata of society do not receive adequate dental care [10, 11].

Interventions in the form of oral health promotion, and education-based strategies, particularly innovative health education approaches, are instrumental in preventing oral diseases and promoting a health culture [12]. By implementing these recommendations dental disease for this age group could be reduced by 80% [13].

Since the 1970s, in the Scandinavian countries, Greece, Portugal, and the United States, preventive public health programs have contributed to a decrease in the prevalence of tooth decay and periodontal diseases for the children of these countries [14, 15]. Over the past two decades, a review of data regarding oral health, sugar consumption, fluoride availability, and preventive programs in 20 selected developed and developing countries has been reported. Among the developed countries, Australia, Denmark, Finland, the Netherlands, New Zealand, Norway, Sweden, the United Kingdom, and the USA, the report indicated a notable decrease (30–50%) in the prevalence of dental caries among 5- and 12-year-old children for past 10 years [14, 15]. The reduction in dental caries among children in developed countries can be attributed to the widespread consumption of fluoridated water, fluoride supplement use, regular use of fluoridated toothpaste, the implementation of preventive oral health services, increased oral health awareness through organized health education programs, and the accessibility of dental resources [15]. Additionally, health promotion initiatives in school settings, including fluoride treatments, examinations, and primary treatments, have also played a role in reducing these oral health disparities [13, 16, 17].

In 2015, an oral health system advancement plan was approved to aim at educating and determining the oral health needs of Iranian school-aged children, by implementing the oral health electronic ID program and planning for preventive services (varnish fluoride). However, this plan’s focus was on the application of varnish fluoride where a limited range of oral health-promoting behaviors were also included. Therefore, the ultimate promotion of positive oral health-behavior plans could be established through expert-led school-based programs in Iran.

Therefore, this study designed, implemented and evaluated an educational intervention to improve the oral health of primary school students. A descriptive-analytical study was conducted among primary school students in Hamedan in 2016 to identify the target group for the intervention and determine the best intervention method. In addition, this descriptive-analytical study determined a conceptual framework based on social cognitive theory to identify factors influencing oral health promotion behaviors using structural equation modelling. An educational intervention was then designed and implemented based on the results of this descriptive-analytical study. The results of the descriptive-analytical study showed that sixth grade girls (11–12 years old) had the highest DMFT index and oral health problems [18, 19]. In addition, no statistically significant differences were found in the prevalence of oral and dental problems between different areas of the city (privileged, semi-privileged and underprivileged). Therefore, sixth grade primary school students (11–12 years old) from all areas of the city were selected as the target group for the intervention. Furthermore, the results of this descriptive-analytical study showed that 50% of the variance in toothbrushing behavior and 55.6% of the variance in flossing behavior were explained by constructs from social cognitive theory, and these models provided a good fit to the data [20]. Therefore, this theory was used to design and implement the intervention. The aim of this study was to evaluate the impact of an oral and dental health promotion education program for primary school children and to present an educational model.

## Methods

The aim of the present study was to determine the effect of oral health education programs on the oral health of primary school students.

### Design and setting

This parallel arm trial design was conducted among fifth-grade elementary school girls in Hamadan, western Iran, between December 2017 and March 2018.

### Process

In this study, the participants were selected using multistage cluster sampling. The Ministry of Education dividesHamadan into two educational regions (region one and region two).

**Fig. 1:**
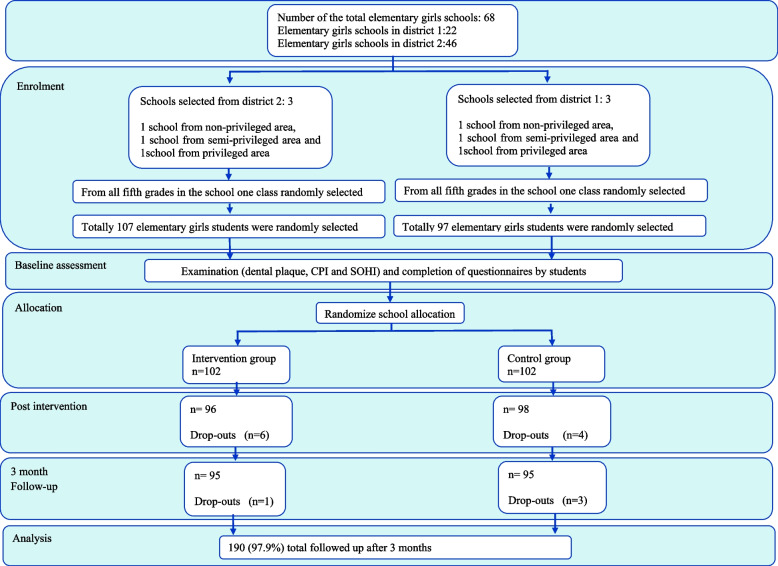
Flow chart of stratify and study subjects through different phases of the trial

The developed interventions were performed in three girls’ elementary schools in Hamadan during the fall and winter of 2017. The participants in the control group received no intervention. Three All-Girls-Schools were identified, and randomized based on the students’ socioeconomic status, representing various educational regions, and the availability of oral health care. Next, a fifth-grade class was selected from school using simple random sampling. Last, the schools were randomly allocated as intervention and control groups after the initial evaluation for the level of plaque, gingival health, and oral health status. All participants completed the pre and post-test questionnaires. In total, 102 students were included and seven were excluded due to incomplete questionnaires and lack of parental cooperation (Fig. 1).

The sample size was determined based on the following formula: (z_1-α/2_ + _z1-β_) 2 (δ_1_^2^ + δ_2_^2^)/d^2^. Based on previous studies, the standard deviation of the plaque index for the two groups was 4.11, which was the same for both groups [21]. The required precision of the estimate (d) was set at 2, and the confidence interval was 95%. A 5% non-response error was then added. A total of 95 students per group were enrolled in the study.

The inclusion criteria included any female student with an age range of 11–12 years, no history of systemic diseases, and no orthodontic treatment at the time of the study.

The exclusion criteria were the presence of oral mucosal lesions, students’ need for any type of dental emergency, absence from more than two educational sessions, incomplete questionnaire (more than 5% missing information), and failure to attend the post-test and examination session after the intervention.

The educational component of this program consisted of one session for mothers and 10 educational sessions for students. During the mother’s educational session, information on the importance of oral health, its role in overall body health, and how to implement the intervention program was presented. The student intervention protocol is explained as the intervention section.

This research considered, the behaviors of brushing twice a day, using fluoride toothpaste, flossing at least once a day, and determination of CPI and OHI-S plaque indexes, as primary outcomes. Bashirian et al. indicated that 50% of brushing behavior and 55.6% of flossing behaviors were explained by the structures of social cognitive theory supported by our study as the ideal model [20].

Therefore, we designed and implemented our interventions on social cognitive theory. The questionnaire related to SCT was used in support of existing validated research by Bashirian et al. [20]. Notably, the questionnaires were completed by the researcher based on conducted interviews.

The students in the intervention and control groups were evaluated in terms of knowledge, structures of SCT, oral health-promoting behaviors (brushing twice a day using a fluoride toothpaste and flossing at least once a day), and oral health indicators (Plaque Index, OHI-S, CPI) before the intervention and 3 months after it.

The interventions and educational materials were designed under the supervision of specialists in health education and promotion (*N* = 2), community dentistry (*N* = 1), and a media expert (*N* = 1). The educational content was prepared using an educational video and booklet entitled “General information of oral health, special for teachers and instructors”, which is the main educational content for students related to the Oral Health Office, Noncontagious Diseases Unit, Deputy Of Health, and Ministry Of Health, Treatment, and Medical Education.

The tailored instructional media training included: seven short educational animations about the structure of teeth, the structure of periodontium, tooth decay, and its stages, periodontal disease, causes of tooth decay and periodontal disease, teaching appropriate brushing technique, use of fluoride-containing toothpaste, and teaching the proper flossing techniques. In addition, three educational pamphlets were produced and used to teach about tooth decay, diseases of periodontal tissues, and preventive methods for tooth decay and periodontal diseases. Additionally, five educational posters were included as teaching tools with topics of “tooth decay”, “gum diseases”, “how to brush your teeth”, “how to floss”, and “some health recommendations.”

The educational tools as prepared media were peer-reviewed and modified accordingly by experts in the field of health education and promotion, community dentists, as well as media experts.

Before the implementation of the interventional program, instructional media was provided to 30 fifth-grade girls’ were provided with instructional media and asked to provide feedback on the media. The media’s problems were identified and eliminated through the students’ feedback.. Moreover, the educational methods of the study were first implemented on a group of 10 fifth-grade girls elementary school students as a trial to identify and eliminate problems before the intervention.

#### Intervention

##### Student educational session

Interventions related to students included displaying videos, playing games, and performing a show for students. Specific intervention strategies to improve student oral health behaviors are listed in Table 1. It is worth noting that the brushing and flossing recording chart was provided to students to remind them to perform oral health behaviors. The researcher evaluated the forms of students in each class once a week to assess the level of adherence to oral health behaviors among students at home.

**Table 1:** Specific intervention strategies to improve oral health behavior in student

Theme	Method	Strategy	Target variable	T	E	P	Participation of student
Session 1. Introducing students to Structure of Teeth and periodontal	displaying film	“Tooth Structure”	Increasing knowledge	1 m	PI	S	
		“Periodontal Structure”	Increasing knowledge	1 m			
	playing games	Dividing students into groups of five, giving colored paper, scissors, colored yarn and liquid glue to groups Students repeatedly looks at different parts of the film and made the replica	Self-Evaluative Outcome -Physical Outcome	60 m	PI	S	Making replica tooth structure and periodontal
	**Telegram group**	Uploaded educational movie for this session Parents are encouraged to buy toothbrushes, fluoride-containing toothpaste and toothpaste for their child Encouraging mothers to monitor their child’s toothbrushing and flossing and encouraging them to behave Encouraging teachers to encourage students to brush and floss	- Situational Perception - Emotional Coping - family& school Environment		PI M	H	Study and review the material at home with the mother
Session 2. Introducing students to Stages of tooth decay	Displaying films	Tooth decay and its stages	Increasing knowledge	2 m	PI	S	
	Playing games	Dividing students into groups of five, giving colored paper, scissors, colored yarn and liquid glue to groups Students repeatedly looks at different parts of the film and made the replica	Self-Evaluative Outcome -Physical Outcome	60 m			Making replica Stages of tooth decay
	Pamphlet	“tooth decay “	Self-Evaluative Outcome -Physical Outcome				
	Installing posters in class	“tooth decay “					
	**Telegram group**	Uploaded movie, pamphlets and educational posters presented at the session Encouraging mothers to monitor their child’s toothbrushing and flossing and encouraging them to behave Encouraging teachers to encourage students to brush and floss	- Situational Perception - Emotional Coping - family& school Environment		PI M	H	Study and review the material at home with the mother
Session 3. Introducing students to Stages of periodontal disease	Displaying films	periodontal disease and its stages	Increasing knowledge	2 m	PI	S	
	Playing games	Dividing students into groups of five, giving colored paper, scissors, colored yarn and liquid glue to groups Students repeatedly looks at different parts of the film and made the replica	- Self-Evaluative Outcome -Physical Outcome	60 m			Making replica Stages of periodontal disease
	Pamphlet	periodontal	Self-Evaluative Outcome -Physical Outcome				
	Installing posters in class	periodontal disease					
	**Telegram group**	Uploaded movie, pamphlets and educational posters presented at the session Encouraging mothers to monitor their child’s toothbrushing and flossing and encouraging them to behave Encouraging teachers to encourage students to brush and floss	- Situational Perception - Emotional Coping - family& school Environment				Study and review the material at home with the mother
Session 4. Introducing students to dental plaque	Displaying films	Causes of tooth decay and periodontal disease	Increasing knowledge	1 m	PI	S	Discuss and discuss with each other
	Group discussion	Discussion about the causes of dental plaque formation and how it causes gum bleeding with the poster of gum disease and dental caries	- Self-Evaluative Outcome - Social Outcome	30 m			
	**Telegram group**	Uploaded movie, pamphlets and educational posters presented at the session Encouraging mothers to monitor their child’s toothbrushing and flossing and encouraging them to behave Encouraging teachers to encourage students to brush and floss	- Situational Perception - Emotional Coping - family& school Environment		PI M	H	Study and review the material at home with the mother
Session 5. Introducing students to ways to prevent dental caries (The proper brushing technique)	Displaying films	- “How to brush and use fluoride-containing toothpaste correctly”	Increasing knowledge	2 m	PI	S	
	Demonstration	- Distribution of toothbrush and toothpaste containing fluoride - educational movie screening step by step and brushing with it - Verbal persuasion - Examination how each students brush by each other - Examination how to brush each student by researcher and correcting problems	- Self Efficacy - Self Efficacy In Overcoming Impediments - Situational Perception - Intention & Behavior	30 m			brushing with fluoride toothpaste
	Installing posters in class	“How to brush”	Increasing knowledge				
	**Telegram group**	Upload video and educational pamphlets presented at this meeting Encourage students to brush twice a day Encourage students to upload their own photos while brushing in group Encouraging mothers to monitor their child’s toothbrushing and flossing and encouraging them to behave Encouraging teachers to encourage students to brush and floss	- Self Efficacy - Self Efficacy In Overcoming Impediments - Situational Perception - Emotional Coping - family& school Environment		PI M	H	Study and review the material at home with the mother -uploded picture
Session 6. Introducing students to ways to prevent dental caries (The proper brushing technique)	Demonstration	- Broadcasted a poem that covered all the objectives of the study in class - Brushing students with music - Examination how to brush each student by researcher and correcting problems	Self Efficacy - Self Efficacy In Overcoming Impediments - Situational Perception - Intention & Behavior	30 m	PI M	S H	
Session 7. Introducing students to ways to prevent dental caries (The proper flossing technique)	Displaying films	- Screening of the two-minute educational movie”How to Use Tooth Floss”	Increasing knowledge	2 m	PI	S	Tooth flossing
	Demonstration	- Distribution of dental floss and toothpaste containing fluoride - educational movie screening step by step and flossing with it - Verbal persuasion - Examination how each students floss by each other - Examination how to floss each student by researcher and correcting problems	Self Efficacy - Self Efficacy In Overcoming Impediments - Situational Perception - Intention & Behavior	30 m			
	Installing posters in class	“How to floss teeth”	Increasing knowledge				
	**Telegram group**	Upload video and educational pamphlets presented at this meeting Encourage students to brush twice a day Encourage students to upload their own photos while brushing in group Encouraging mothers to monitor their child’s toothbrushing and flossing and encouraging them to behave Encouraging teachers to encourage students to brush and floss	- Self Efficacy - Self Efficacy In Overcoming Impediments - Situational Perception -Emotional Coping - family& school Environment		PI M	H	Study and review the material at home with the mother -uploded picture
Session 8. Introducing students to ways to prevent dental caries (The proper flossing technique)	Demonstration	- Broadcasted a poem that covered all the objectives of the study in class - flossing students with music - Examination how to floss each student by researcher and correcting problems	- Self Efficacy - Self Efficacy In Overcoming Impediments - Situational Perception - Intention &Behavior	30 minute	PI	S	Tooth flossing
	Pamphlet	“How to have healthy and beautiful teeth” Explain the necessity of flossing once a day	- Self Efficacy In Overcoming Impediments - Intention				
	Installing posters in class	“A few health tips”	- Intention				
	**Telegram group**	Upload video and educational pamphlets presented at this meeting Encourage students to brush twice a day Encourage students to upload their own photos while brushing in group Encouraging mothers to monitor their child’s toothbrushing and flossing and encouraging them to behave Encouraging teachers to encourage students to brush and floss	- Self Efficacy - Self Efficacy In Overcoming Impediments - Situational Perception - Emotional Coping - family& school Environment		PI M	H	Study and review the material at home with the mother -uploded picture
Session 9. Introducing students to ways to prevent dental caries	Demonstration	- Playing poetry and brushing and flossing students with poetry and correcting their problems	- Self Efficacy	30 m	PI	S	Brushing, flossing in class, Role playing
	**Role Playing**	- Divide students into groups of 5 or 6, and write group exercises together on dental caries and dental caries diseases and ways to prevent dental caries.	- Self-Evaluative Outcome -Physical Outcome - Social Outcome -Self Efficacy - Self Efficacy In Overcoming Impediments - Situational Perception - Intention &Behavior	60 m			
	**Telegram group**	Upload video and educational pamphlets presented at this meeting Encourage students to brush twice a day Encourage students to upload their own photos while brushing in group Encouraging mothers to monitor their child’s toothbrushing and flossing and encouraging them to behave Encouraging teachers to encourage students to brush and floss	- Self Efficacy - Self Efficacy In Overcoming Impediments - Situational Perception - Emotional Coping - family& school Environment		PI M	H	Study and review the material at home with the mother -uploded picture
Session 10. Introducing students to ways to prevent dental caries	Demonstration	- Playing poetry and brushing and flossing students with poetry and correcting their problems	-Self Efficacy	30 m	PI	S	Brushing, flossing in class, Role playing
	**Role Playing**	-Role Playing - Students commented on the strengths and weaknesses of the plays and discussed the topics discussed in the plays and their drawbacks.	- Self-Evaluative Outcome -Physical Outcome - Social Outcome -Self Efficacy - Self Efficacy In Overcoming Impediments - Situational Perception - Intention &Behavior	60 m			
	**Telegram group**	Encourage students to brush twice a day Uploading student performance videos Upload video and educational pamphlets presented at this meeting Encourage students to upload their own photos while brushing in group Encouraging mothers to monitor their child’s toothbrushing and flossing and encouraging them to behave Encouraging teachers to encourage students to brush and floss	- Self Efficacy - Self Efficacy In Overcoming Impediments - Situational Perception - Emotional Coping - family& school Environment		PI M	H	Study and review the material at home with the mother -uploded picture

*T* Time, *P* Place, *E* Educator, *PI* Principal investigator, *M* Mother, *S* School, *H* Home

##### Telegram group

A Telegram group entitled “Oral Health” was created and joined by students’ family members, teachers, and administrators from each school, as well as the community dentist and health education professor. The educational videos were shown at each session, oral health educational content was posted to the group, members’ oral health questions were answered, and students were encouraged to take pictures of themselves during brushing and flossing and post them to the group.

##### Dental health ceremony

The educational intervention concluded with an oral health ceremony at each school attended by the students’ parents. During the ceremony, the students performed their shows for their parents, as well as their principal and teachers.

##### Follow-up and post-test

In the follow-up course, which was conducted for 3 months after the final educational session, educational videos, pictures, and oral health-related content were posted to the Telegram group. Every 2 weeks, the researcher contacted the schools to observe the students’ brushing and flossing and to point out their mistakes. In the end, five students who brushed and flossed properly were recognized. Moreover, these individuals were asked to help identify other students’ mistakes. A post-test was administered to both groups at the end of the follow-up period.

### Statistical analysis

The statistical analysis was performed using SPSS version 16.0 software. Descriptive statistics and parametrical tests (e.g., analysis of variance, independent t-test, and paired t-test) were used to determine the significant statistical differences between the groups and compare them in terms of quantitative variables. Furthermore, Chi-square, Kappa’s test, and McNemar’s test were also used to evaluate the qualitative variables.

## Results

In this study, 77.8 and 82.1% of students in the control and intervention groups experienced a toothache in the past year, respectively. On the other hand, 34.7 and 26.3% of the participants in the control and intervention groups resided in the outskirts, respectively. However, no significant difference was observed between the research groups in terms of demographic variables. In this study, there were no important adverse events.

### Oral health behavior

According to the results, there was an insignificant difference in the control group regarding the number of daily brushings before and 3 months after the intervention (*P* = 0.82). Meanwhile, a significant difference was observed in the intervention group (*P* <  0.001, Table 2). There was also a significant difference was detected between the intervention and control groups in the number of brushings per day after the intervention (*P* <  0.001, Table 2). In the intervention group, brushing two or more times per day increased from 10.5 to 59%. Brushing every two or 3 days (often) decreased from 32.6 to 8.4%. In the control group, 17.9% of participants brushed their teeth two or more times per day before and after the intervention (Table 2).

**Table 2:** Distribution of oral health behaviors and Oral Health Indicators before and 3 months after intervention in the two groups

Behavior	Groups			After				Total	*P*_*value*_	
				Never	Often	Once	Twice or more			
Tooth brushing frequency per day	Before	Intervention	Never	0(0.0)	3(3.2)	0(0.0)	1(1.0)	4(4.2)	< 0.001^a^	< 0.001^b^
			Often	0(0.0)	3(3.2)	8(8.4)	20(21.0)	31(32.6)		
			Once	0(0.0)	2(2.1)	20(21.1)	28(29.5)	50(52.6)		
			Twice or more	0(0.0)	0(0.0)	3(3.2)	7(6.4)	10(10.5)		
			Total	0(0.0)	8(8.4)	31(32.7)	56(59.0)			
		Control	Never	3(3.2)	3(3.2)	1(1.0)	1(1.0)	8(8.4)	0.82 ^a^	
			Often	1(1.0)	11.(11.5)	12.(12.5)	3(3.2)	27(28.4)		
			Once	0(0.0)	2(2.1)	34(35.8)	7(7.4)	43(45.3)		
			Twice or more	0(0.0)	5(5.3)	5(5.3)	7(7.4)	17(17.9)		
			Total	4(4.2)	21(22.1)	52(54.6)	18(19)			
	***P***_***value***_	0.54 ^b^								
				After				Total	***P***_***value***_	
				No brushing	< 1 min	1–2 min	≥2 min			
Brushing time*	Before	Intervention	No brushing	0(0.0)	1(1.0)	0(0.0)	3(3.2)	4(4.2)	< 0.001^c^	0.21 ^b^
			< 1 min	0(0.0)	2(2.1)	1(1.0)	15(15.8)	18(18.9)		
			1–2 min	0(0.0)	1(1.0)	3(3.2)	30(31.5)	34(35.8)		
			> 2 min	0(0.0)	2(2.1)	2(2.1)	35(36.8)	39(41.1)		
			Total	0(0.0)	6(6.3)	6(6.3)	83(87.3)			
		Control	No brushing	3(3.2)	1(1.0)	1(1.0)	3(3.2)	8(8.4)	0.11^c^	
			< 1 min	1(1.0)	3(3.2)	3(3.2)	15(15.8)	22(23.2)		
			1–2 min	0(0.0)	4(4.2)	5(5.3)	13(13.7)	22(23.2)		
			> 2 min	0(0.0)	13(13.7)	6(6.3)	24(25.2)	43(45.3)		
			Total	4(4.2)	21(22.1)	15(18.8)	55(57.9)			
	***P***_***value***_		< 0.001 ^b^							
					After			Total	***P***_***value***_	
					Never	Often	Once or more			
Tooth flossing frequency per day	Before	Intervention	Never		4(4.2)	8(8.4)	38(39.9)	50(60.0)	< 0.001^a^	0.02 ^b^
			Often		0(0.0)	9(9.4)	26(27.3)	35(36.8)		
			Once or more		1(1.0)	2(2.1)	7(7.4)	10(10.5)		
			Total		5(5.3)	19(20.0)	71(74.7)			
		Control	Never		28(29.5)	5(5.3)	7(7.4)	40(42.1)	0.52 ^a^	
			Often		14(14.7)	8(8.4)	6(6.3)	28(29.5)		
			Once or more		5(5.3)	4(4.2)	18(19)	27(28.5)		
			Total		47(49.5)	17(17.9)	31(32.7)			
	***P***_***value***_		< 0.001 ^b^							
Indices	Groups				After			Total	***P***_***value***_	
					Healthy	Bleeding	Calculus			
Community Periodontal Index (CPI)	Before	Intervention	Healthy		82(86.3)	1(1.1)	3(3.2)	86(90.5)	0.13^a^	0.007^b^
			Bleeding		7(7.4)	0(0.0)	0(0.0)	7(7.4)		
			Calculus		1(1.1)	0(0.0)	1(1.1)	2(2.1)		
			Total		90 (94.7)	1(1.1)	4(4.2)			
		Control	Healthy		91(95.7)	0(0.0)	2(2.1)	93(97.9)	1^a^	
			Bleeding		0(0.0)	0(0.0)	0(0.0)	0(0.0)		
			Calculus		1(1.1)	0(0.0)	1(1.1)	2(2.1)		
			Total		92(96.8)	0(0.0)	3(3.2)			
	***P***_***value***_				0.46 ^b^					
					After			Total	***P***_***value***_	
					Good	Fair	poor			
Simplified Oral Hygiene Index (OHI-S)**	Before	Intervention	Good		32(33.6)	4(4.2)	0(0.0)	36(37.9)	< 0.001^c^	0.03^b^
			Fair		40(42.1)	12(12.6)	0(0.0)	52(54.7)		
			poor		6(6.3)	1(1.1)	0(0.0)	7(7.4)		
			Total		78(82.1)	17(17.9)	0(0.0)			
		Control	Good		19(20.0)	1(1.1)	0(0.0)	20(21.1)	< 0.001^c^	
			Fair		25(26,3)	44(46.3)	0(0.0)	69(72.6)		
			poor		1(1.1)	2(2.1)	3(3.2)	6(6.3)		
			Total		45(47.4)	47(49.5)	3(3.2)			
	***P***_***value***_				< 0.001 ^b^					
					intervention	Control		***P***_***value***_		
					Mean (SD)	Mean (SD)				
Plaque index (PI)			Before		67.78(29.74)	64.57(26.65)		0.43^d^		
			After		31.60(24.32)	57.29(27.95)		< 0.001^d^		
			Difference		36.14	7.27		< 0.001^d^		
			***P***_***value***_		< 0.001^e^	0.01 ^e^				

^a.^ Kappa test

^b.^ Chi-square

^c^. McNemar

d. Independent Samples T Test,

e. Paired samples T test

*The Brushing time was further recoded to < 2 min and ≥ 2 min

**The oral hygiene status was further recoded to good oral hygiene and fair/poor oral hygiene

In addition, there was an insignificant difference in the control group regarding the duration of brushing before and 3 months after the intervention (*P* = 0.11), whereas a significant difference was observed in the intervention group (*P* <  0.001, Table 2). There was also a significant difference in this behavior between the intervention and control groups (*P* <  0.001, Table 2). In the intervention group, brushing for 2 minutes or more increased from 41.2 to 87.3% (Table 2).

The results also showed an insignificant difference in the control group in the number of times that participants flossed per day before and 3 months after the intervention (*P* = 0.52). However, a statistically significant difference was observed in the intervention group in this regard (*P* <  0.001). Notably, a significant difference was found between the intervention and control groups regarding the mentioned behavior (*P* <  0.001, Table 2). Before the intervention, 28.5 and 10.5% of students in the control and intervention groups flossed one or more times a day, respectively, which reached 32.7 and 74.7% in the aforementioned groups after the intervention, respectively (Table 2).

The outcome of the knowledge and social cognitive theory questionnaire:

Initially, an insignificant difference was noticed between the intervention and control groups as the mean knowledge score (*P* = 0.41), however, upon the completion of the interventions that significance was notable, (*P* <  0.001, Table 3).

**Table 3:** Differences in dependent variables before and 3 months after intervention in the two groups

Behavior	Variable	Group	Control	Intervention	*P*_value_^a^	Re-range Scores^c^		
			Mean (SD)	Mean (SD)		Control	Intervention	Difference
Knowledge		Before	2.56(1.39)	2.41(1.23)	0.41	36.5	34.7	1.8
		After	3.05(1.50)	5/50(1.21)	<0.001	43.5	78.5	35
		Difference	0.48	3.09	<0.001	7	43.8	
		P _value_^b^	0.02	<0.001				
Brushing with fluoride toothpaste	Behavior	Before	5.67(1.30)	5.26(0.90)	0.01	44.5	36.6	7.9
		After	5.76(1.17)	7.16(1.12)	<0.001	46.0	69.3	22.1
		Difference	0.10	1.90	<0.001	1.5	32.7	
		P _value_^b^	0.83	<0.001				
	Self Efficacy	Before	4.59(1.07)	4.07(0.89)	<0.001	64.7	51.7	13
		After	4.55(1.11)	5.30(1.83)	<0.001	63.7	82.5	18.8
		Difference	−0/03	1.23	<0.001	1	30.8	
		P _value_^b^	0.33	<0.001				
	Environment (school)	Before	5.14(2.04)	4.57(1.31)	0.02	14.2	7.2	7/0
		After	4.32(0.83)	6.86(2.80)	<0.001	4	35.7	31.7
		Difference	−0.82	2.29	<0.001	10.2	28.5	
		P _value_^b^	<0.001	<0.001				
	Intention	Before	7.83(1.41)	7.20(1.11)	0.001	80.5	70	10.5
		After	7.78(1.50)	8.48(0.90)	<0.001	79.6	91.3	11.7
		Difference	−0.04	1.28	<0.001	0.9	21.3	
		P _value_^b^	0.83	< 0.001				
	Physical Outcome	Before	6.82(1.66)	6.69(1.63)	0.59	63.6	61.5	2.1
		After	7.06(1.87)	7.82(1.42)	0.002	79.5	81.3	1.8
		Difference	0.24	1.12	0.002	15.9	19.8	
		P _value_^b^	0.26	< 0.001				
	Self Efficacy In Overcoming Impediments	Before	5.94(2.05)	5.72(1.69)	0.42	49	45.3	3.6
		After	6.03(1.99)	6.87(1.51)	0.001	50.5	64.5	14
		Difference	0.08	1.14	<0.001	1.5	19.2	
		P _value_^b^	0.70	< 0.001				
	Emotional Coping	Before	9.44(1.77)	9.00(1.54)	0.06	68	62.5	5.5
		After	10.36(1.97)	10.51(1.56)	<0.001	79.5	81.3	1.8
		Difference	−0.07	1.15	<0.001	11.5	18.8	
		P _value_^b^	0.74	< 0.001				
	Outcome Expectations	Before	27.89(4.29)	26.77(3.93)	0.06	76.3	71.6	4.7
		After	29.36(3.46)	30.01(2.99)	0.17	83.4	86.4	3
		Difference	1.47	3.24	0.01	7.1	14.8	
		P _value_^b^	0.003	<0.001				
	Environment (family)	Before	18.06(3.98)	17.52(3.30)	0.31	62.5	59.5	3
		After	18.20(3.68)	19.53(3.35)	0.22	63.7	72	8.2
		Difference	0.14	2.01	0.04	1.2	12.5	
		P _value_^b^	0.06	<0.001				
	Situational Perception	Before	3.29(1.24)	3.18(1.07)	0.53	32.2	29.5	2.7
		After	3.17(1.06)	3.58(1.31)	0.01	29.2	39.5	10.3
		Difference	−0.11	0.40	0.01	3	10	
		P _value_^┼^	0.43	0.008				
	Perceived Barriers	Before	9.54(1.88)	9.16(1.63)	0.14	69.2	64.5	4.7
		After	9.22(2.02)	9.94(1.63)	0.007	65.2	74.2	9
		Difference	−0.32	0.77	0.001	4	9.7	
		P _value_^b^	0.14	0.002				
	Outcome Expectancies	Before	26.88(3.88)	25.62(3.44)	0.01	84.4	78.1	6.3
		After	26.52(3.75)	27.38(2.87)	0.07	82.6	86.9	4.3
		Difference	−0.35	1.76	0.001	1.8	8.8	
		P _value_^b^	0.47	<0.001				
Flossing	Self Efficacy	Before	7.33(2.56)	6.10(2.02)	<0.001	41.6	26.5	15.1
		After	7.40(2.66)	9.53(1.91)	<0.001	42.5	69.1	26.6
		Difference	−0.76	3.43	<0.001	0.9	42.6	
		P _value_^b^	0.09	<0.001				
	Behavior	Before	3.77(1.29)	3.18(0/62)	<0.001		44.2	29.5
		After	3.50(1.03)	4.87(1.15)	<0.001		37.5	71.7
		Difference	−0.27	1.68	<0.001		6.2	42.2
		P _value_^b^	0.03	<0.001				
Flossing	Outcome Expectations	Before	21.36(5.59)	18.35(4.82)	<0.001	56.8	41.7	15.1
		After	21.47(6.04)	25.54(4.27)	<0.001	57.3	77.5	20.2
		Difference	0.11	7.18	<0.001	0.5	35.8	
		P _value_^b^	0.86	< 0.001				
	Environment (family)	Before	9.71(3.33)	8.36(2.56)	0.002	47.1	33.6	13.5
		After	9.01(3.09)	11.75(2.32)	<0.001	40.1	67.5	27.4
		Difference	−0.70	3.38	<0.001	7	33.9	
		P _value_^b^	0.06	<0.001				
	Intention	Before	2.76(0.49)	1.53(0.72)	<0.001	88.0	26.5	13.5
		After	1.99(0.88)	2.16(0.81)	< 0.001	49.5	58.0	27.4
		Difference	−0.24	1.23	< 0.001	38.5	31.5	
		P _value_^b^	0.02	<0.001				
	Environment (school)	Before	4.89(1.67)	4.33(1.05)	0.007	11.1	4.12	6.9
		After	4.27(1.05)	6.80(2.75)	<0.001	3.37	35.0	28.3
		Difference	−0.62	2.46	<0.001	7.7	30.8	
		P _value_^b^	0.001	<0.001				
	Emotional Coping	Before	8.80(1.97)	8.03(1.52)	0.003	60.0	50.3	9.7
		After	8.78(1.91)	10.31(1.61)	<0.001	59.7	78.8	19.1
		Difference	−0.02	2.48	<0.001	0.3	28.5	
		P _value_^b^	0.96	<0.001				
	Situational Perception	Before	3.06(1.08)	2.66(0.94)	0.007	26.5	16.5	10
		After	3.02(0.94)	3.54(1.22)	0.001	25.5	38.5	13
		Difference	−0.04	0.88	<0.001	1	22	
		P _value_^b^	0.73	<0.001				
	Perceived Barriers	Before	9.64(1.75)	9.17(1.76)	0.07	70.5	64.6	5.4
		After	9.83(1.82)	10.35(1.50)	0.03	72.8	79.3	6.5
		Difference	0.18	1.17	0.003	2.3	14.7	
		P _value_^b^	0.45	<0.001				
	Outcome Expectancies	Before	26.88(3.88)	25.62(3.44)	0.01	84.4	78.1	6.3
		After	26.52(3.75)	27.38(2.87)	0.07	82.6	86.9	4.3
		Difference	−0.35	1.76	0.01	1.8	8.8	
		P _value_^b^	0.47	<0.001				

^a^. Independent Samples T Test

^b^. paired sample T test

^c^. The scores between two groups, I.e., intervention and control groups, re-change to 0–100 for analysis

In other words, the mean knowledge score increased significantly in the control (7%) and intervention (43.8%) groups after the intervention (*P* <  0.05, Table 3). In addition, a significant difference was found between the intervention and control groups in the mean scores of all SCT concepts related to brushing with fluoride toothpaste and flossing among students (*P* <  0.05, Table 3). In the brushing model, most of the changes after the intervention were observed in participants’ behavior (32.7% increase) and self-efficacy (30.8% increase),while the least changes were observed in perceived barriers (9.7% increase) and value expectancy (8.8% increase, Table 3). In the flossing model, the largest changes were related to self-efficacy (42.6% increase) and behavior (42.2% increase), while the smallest changes were related to perceived barriers (14.7% increase) and value expectancy (8.8% increase, Table 3).

### Oral health indicators

The results indicated no significant difference between the control (*P* = 1) and intervention (*P* = 0.13) groups in terms of CPI before and 3 months after the intervention (Table 2). In addition, while a significant difference was observed between the intervention and control groups before the intervention (*P* = 0.007), no significant difference was observed between the groups after the intervention (*P* = 0.46, Table 2). Before the intervention, 97.9% of students in the control group had healthy gums, and 100% had no bleeding gums. In the intervention group, healthy gums without bleeding were observed in 90.5 and 7.4% of the participants, respectively. After the intervention, there was a 1.1% decrease in healthy gums in the control group and a 4.2% increase in the intervention group. Moreover, bleeding gums decreased by 6.3% in the intervention group (Table 2).

According to the results of the study, there was a significant difference between the control group (*P* <  0.001) and the intervention group (*P* <  0.001) with regard to the OHI-S indicator before and 3 months after the intervention. Threr was also a significant difference between the intervention and control groups before (*P* = 0.003) and 3 months after the intervention(*P* <  0.001). In the intervention group, the OHI-S indicator was reported as good by 37.9% of the intervention students before the intervention, which increased to 82.1% after the intervention. While an insignificant difference was observed between the intervention and control groups in the mean plaque index score before the intervention (*P* = 0.43), the difference between the groups became significant after the intervention (*P* <  0.001). In general, the plaque index decreased significantly in both groups after the intervention (*P* <  0.05, Table 2).

## Discussion

purpose of this study was to evaluate the effectiveness of an intervention designed to prevent dental plaque and periodontal disease in elementary school students.

According to the results of the present study, the intervention significantly changed all structures of the social cognitive theory in both behaviors of brushing with fluoride toothpaste and flossing in the participants of the intervention and control groups after the intervention. Finally, most of the differences observed in the students’ scores before and after the intervention were related to self-efficacy, behavior, outcome expectancy, school environment, intention, emotional coping, family environment, situational understanding, perceived barriers, and value expectancy.

The interventions had a significant impact on the increase in students’ knowledge. The results of similar intervention studies conducted in this area using different strategies [13, 16, 17] have demonstrated the effectiveness of education in improving students’ oral health knowledge. The reason for the increase in knowledge in the intervention group is that in this group the education was based on the game, role-playing and demonstrations, and during the three-month follow-up period the students were not left alone and received educational videos and pamphlets via Telegram. In the control group, the average knowledge of the students before and after the intervention was statistically significant. The reason for the increase in knowledge in the control group is related to the implementation of the Oral Health Transformation Plan, which was carried out concurrently with the interventions of this study by the Ministry of Health at the school level throughout the country. In this program, schools are visited every 6 months to apply fluoride varnish to students, in addition to teaching them the correct way to brush their teeth.

According to the results, the students’ self-efficacy variable in students ranked first in terms of the level of change before and after the intervention. This significant increase in self-efficacy of the participants in the intervention group, compared to the control group, might be due to the use of all self-efficacy enhancing strategies (e.g., considering stage goals, improvement, and control, education to control negative emotional responses, providing verbal persuasion, enhancing the adoption and maintenance of behavior, and creating behavioral patterns in students) in the design and implementation of interventions. Consistent with our findings, the results of various studies have indicated the effectiveness of detailed interventions in promoting self-efficacy in individuals regarding oral health-promoting behaviors [22, 23].

The second concept of social cognitive theory that changed the most after the intervention was outcome expectancy, which is influenced by the behavior of people students see or receive awards from.. In the present study, education was provided both through observation (education by classmates and observation of classmates’ behavior in the Telegram group) and through the necessary persuasion of family members, teachers, the researcher, and classmates. Most studies have highlighted the predictive role of outcome expectancy on oral health-promoting behaviors and the effectiveness of interventions in promoting outcome expectancy status in participants [24, 25].

School environment was the third concept of social cognitive theory that showed the most change in students after the intervention. The World Health Organization declares that the school is one of the most effective variables in promoting oral health, and teaching the principles of oral care to students by health educators by providing attractive and informative programs and activities in schools along with increasing the awareness of parents regarding the methods of preventing dental caries and considering incentives from the school to motivate students, it can improve oral health indicators in students and reduce dental treatment costs [26, 27]. The interventions implemented in this study met all the requirements of the World Health Organization and provided the necessary support to the students.

According to the results of the present study, the family environment variable had a low priority in terms of change after the intervention. The impact of the family on children’s oral health has been fully recognized [28, 29]. In the present study, the interventions delivered to families (mostly mothers) included educational content presented via Telegram. Considering the effect of parents’ oral health behaviors and their oral health literacy on children’s oral health behaviors [30–32] and the importance of the family’s role in performing behaviors related to children’s oral health, there is a need for more in-depth interventions to improve families’ oral health skills, attitudes, and oral health literacy.

According to the results of the present study, there was a significant difference between the intervention and control groups in the oral health behaviors of the students. Several studies have shown the effectiveness of interventions in promoting oral health behaviors among students [13, 33]. According to the structural equation model, 55.6% of the variance in flossing and 50% of the variance in brushing were explained by the social cognitive theory structures [20]. Therefore, it could be concluded that improving these structures will lead to an improvement in students’ oral health behaviors. On the other hand, while the participants in the control group also received an education, both brushing and flossing behaviors decreased among students in this group 3 months after the intervention. One of the reasons for this decrease may be that students in the control group received education only once, while in the intervention group, the educator visited the schools once a week to practice brushing and flossing and to solve students’ problems in these areas. In this way, the students received a continuous educational program for 5 months. As the results of the studies show, the greater the distance between the interventions, the intervention will not be effective and its effects are limited only to the time of implementation of the educational program [12]. Another reason for this decrease in the control group may be lectured-based education, as studies have shown that while this technique increases knowledge, it does not have a positive effect on students’ oral health behaviors [12, 34]. Teaching through lectures is not very attractive to students and does not significantly improve their behavior [35]. In the intervention group, the health-related messages were delivered interactively using short shows, simple language, colorful pictures, handicrafts, exercises, and videos. In this group, students were able to receive useful information in a simple and interesting way. In addition, the participants were willing to find new educational materials. In this respect, our results are consistent with those of other studies [13, 36–38].

According to the study results, the interventions affected the plaque index, OHI-S, and CPI. Several studies conducted in different societies have shown the effectiveness of oral health education in reducing dental plaque and improving oral health in students [38–40]. Studies show that brushing and flossing are simple and effective ways to control dental plaque and improve oral health [41]. Therefore, plaque reduction and improved oral health are expected to result from an increase in the health behaviors of brushing and flossing.

However, the change in the level of CPI of the participants in the intervention group was not at the favorable and expected level, while gingival bleeding decreased in the students in the intervention group, and there was no change in the calculus index in the mentioned group. In several studies, no improvement in the gingival index of the participants was observed after educational interventions [42, 43]. Low gum health improvement may be affected by the normal age of puberty and the transition from the mixed dentition to the permanent dentition [42]. In addition, while short-term educational programs improved oral health, they had little impact effect on improving gum status. On the other hand, there may have been no improvement in the gum status in the intervention group before and after the intervention because the students’ gums were healthy at the beginning of the study.

One of the major limitations of the current research was the large number of questionnaire items due to the evaluation of all concepts of social cognitive theory. Completing the the questionnaire was relatively tedious for the students, which may have affected the quality of the data collected. Another limitation was the fact that our study coincided with the implementation of the National Oral Health Plan. However, it is noteworthy that the issue also affected the control group.

However, the present study also had some positive aspects. One of the major strengths of the present study was the use of games as a teaching technique. In addition, social cognitive theory was used in the present study to plan and modify the factors that influence students’ behavior, which is another strength of the present study.

In addition, sampling by a three-stage random strategy resulted in the selection of students in public and private schools in different districts of the city. It is notable that the high response rate and the examination by one person increased the internal validity of the study.

## Conclusion

According to the results of the present study, the intervention implemented was necessary to improve health-promoting behaviors and increase oral health indexes among students. When presented in the form of pamphlets, videos, games, and other educational methods, health-related messages can change children’s behavior and improve their attitudes toward oral health. Gamification as an alternative, useful educational approach can increase knowledge retention in improving oral health for school-aged children. That games can be applied as an alternative strategy to improve oral health in students.

## Data Availability

The author confirms that all data generated or analyzed during this study are included in this published article.

## Abbreviations

SCT: social cognitive theory
DMFT: Decayed, Missing, and Filled Teeth
OHI-S: Simplified Oral Hygiene Index
CPI: Community Periodontal Index
PI: Plaque indexes
WHO: World Health Organization
ADA: American Dental Association
